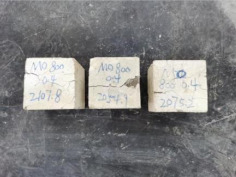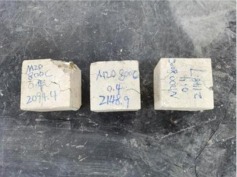# High Temperature Degradation Mechanism of Concrete with Plastering Layer

**DOI:** 10.3390/ma15020398

**Published:** 2022-01-06

**Authors:** Chihao Liu, Jiajian Chen

**Affiliations:** Department of Civil Engineering, Foshan University, Foshan 528001, China; 2111961001@stu.fosu.edu.cn

**Keywords:** high temperature, mechanical properties, plastering layer, water/cement ratio

## Abstract

At present, the research on the high temperature degradation of concrete usually focuses on only the degradation of concrete itself without considering the effect of the plastering layer. It is necessary to take into account the influence of the plastering layer on the high temperature degradation of concrete. With an increase in the water/cement ratio, the explosion of concrete disappeared. Although increasing the water/cement ratio can alleviate the cracking of concrete due to lower pressure, it leads to a decrease in the mechanical properties of concrete after heating. It is proved that besides the water/cement ratio, the apparent phenomena and mechanical properties of concrete at high temperature can be affected by the plastering layer. The plastering layer can relieve the high temperature cracking of concrete, and even inhibit the high temperature explosion of concrete with 0.30 water/cement ratio. By means of an XRD test, scanning electron microscope test and thermogravimetric analysis, it is found that the plastering layer can promote the rehydration of unhydrated cement particles of 0.30 water/cement ratio concrete at high temperature and then promote the mechanical properties of concrete at 400 °C. However, the plastering layer accelerated the thermal decomposition of C-S-H gel of concrete with a water/cement ratio of 0.40 at high temperature, and finally accelerate the decline of mechanical property of concrete. To conclude, the low water/cement ratio and plastering layer can delay the deterioration of concrete at high temperature.

## 1. Introduction

As a stable and reliable building material, concrete is widely used in various infrastructure construction facilities. The service life of concrete can theoretically reach a century under normal service conditions, but concrete can deteriorate rapidly in some extremely harsh environments, such as fire conditions. The Fire and Rescue Bureau of the Ministry of Emergency Management, PRC reported 252,000 fires across China in 2020, resulting in 1183 deaths, 775 injuries and 4.09 billion yuan in direct property losses [1]. Researchers have made many attempts to explore the degradation mechanism of concrete under high temperature. The high temperature degradation of concrete includes strength loss, cracking, spalling and explosion.

The reasons for concrete degradation at high temperature are as follows: high temperature vapor pressure, temperature stress, difference in thermal expansion coefficient between aggregate and paste and thermal decomposition of hydration products [2,3,4,5,6,7], among which the main reasons for concrete degradation are high temperature vapor pressure and thermal decomposition of hydration products. Under 400 °C, the hydration products of concrete do not obviously decompose, the deterioration of concrete is mainly due to the high temperature vapor pressure; when the temperature exceeds 400 °C, the calcium hydroxide (CH), C-S-H gel and calcium carbonate (CaCO_3_) of concrete decompose, resulting in a decline of concrete strength [8,9,10]. Compared with the degradation of concrete caused by thermal decomposition of hydration products, the degradation caused by high temperature vapor and thermal stress is often more serious, because the accumulation of high temperature vapor, to a certain extent, will instantly destroy concrete and cause it to lose all load capacity, ultimately leading to the failure of concrete structure.

The main causes of concrete cracking and explosion are too-low water/cement ratio (especially UHPC [11,12,13,14]) and too-high water content [4]. However, in practical engineering, as concrete cannot have high water content after air drying for several months or even several years, the influence of water/cement ratio on concrete high temperature cracking was only considered in tests.

In actual engineering, the surface of a concrete structure is covered with a layer of plastering mortar to protect the concrete from erosion and provide a decorative effect [15,16]. Under fire, the plastering layer can act as a barrier to decelerate the change of temperate of the internal concrete structure. Therefore, the plastering layer can theoretically delay the deterioration of concrete caused by high temperature vapor pressure (especially low water/cement ratio concrete).

Up to now, there are many researchers who have analyzed the degradation mechanism properties of concrete components (beam, slab and column) with a plaster layer under high temperature by combining a scale structure test and finite element software. Hodhod indicated that traditional cement coating can delay the temperature rise of the RC column, and that traditional mortar coating can increase the residual load capacity of the RC column after 30 min at 650 °C [17]. Andrii reported that a fire-retardant coating can effectively improve the fire resistance of RC floor, and the fire resistance of RC floor increases with the thickness of fire-retardant coating [18]. Allam found through calculation that a plastering layer could delay the heating rate of one-way reinforced concrete slab and the high temperature deterioration of reinforcement and concrete, and finally improved the fire resistance limit of one-way reinforced concrete slab [19]. Carlos found that the beams with passive fire protection materials provided an integrity of their resistance for long periods of fire exposure [20].

The ultimate load capacity and fire resistance of concrete components under fire are determined by the mechanical properties of concrete at high temperature. Du pointed out that the ultimate limit state in fire for the concrete slab under bending moment and compressive force was decided by the mechanical property of concrete [21]. Allam reported that the moment capacity of one-way reinforced concrete slab in fire is provided by reinforcement and concrete [19]. Balaji mentioned that the axial capacity of reinforced concrete columns at high temperature is provided by reinforcement and concrete [22]. At high temperature, the performance of concrete will deteriorate in varying degrees. As the ultimate load capacity of concrete component is provided, with the rise of temperature, the deflection of concrete components increases, and the load capacity decreases until it is completely invalid. Therefore, it is necessary to explore the fundamental mechanism of the mechanical properties of concrete at high temperatures.

In the past studies, researchers generally calculated the fire resistance and moment/axial capacity of concrete members at high temperatures by means of the mechanical strength reduction factor of the reinforcement and concrete. However, the mechanical properties of concrete do not necessarily decrease monotonously with the temperature increase (≤400 °C), and the fundamental mechanisms of concrete mechanical properties under high temperatures are affected by many factors such as water/cement ratio and plaster layer due to the rehydration of cement particles. Simply using the reduction factor cannot adequately calculate the mechanical properties of concrete at high temperatures, which may lead to errors in subsequent calculation and modeling.

Hence, it is necessary to study concrete damage mechanisms under the action of plaster layer, water/cement ratio and high temperature coupling. This paper analyzed the concrete degradation mechanisms of concrete under the coupling effect of water/cement ratio, plastering layer and high temperature by measuring the residual mass of concrete, ultrasonic pulse velocity, compressive strength, XRD diffractograms, SEM images and Thermogravimetric analysis.

## 2. Materials and Methods

### 2.1. Test Procedure

To study the mechanism of the coupling effect of plastering layer and water/cement ratio on heat degradation, a total of 30 groups of concrete specimens with different mortar plaster thickness and different water/cement ratio heated to different temperatures were produced. The paste volume and fine-to-aggregate ratio of all concrete mixtures was set at 0.40 and 0.45, respectively. The water/cement ratio varied from 0.30 to 0.40 with a difference of 0.05. According to the general practice of concrete production, a water reducing agent was added into each concrete mixture to make the workability meet the requirements. Water reducing agent was added to each mixture bit by bit until the mixture had a slump of 100 ± 20 mm. Concrete specimens were prepared according to the standard for evaluation of concrete compressive strength (GB/T50107-2010, China) and for the uniaxial compressive strength test. The size of the concrete sample was 100 × 100 × 100 mm^3^, and each sample was poured in the mold at one time and placed on the vibration table to vibrate evenly. After the specimen was placed at room temperature for 24 h, the mold was removed, and the concrete specimens were soaked in a water tank for 28 d curing. For easy identification, each plastered concrete mixture was assigned a label in the format of water/cement ratio—mortar plastered layer thickness—room temperature/heating temperature. The concrete mixtures are shown in Table 1.

Mortar for plastering was designed in accordance with the Specification for mix proportion design of masonry mortar (JGJ/T98-2010, China). The mixture of mortar is shown in Table 2. Mortar plastering layer thickness varied between 0 and 20 mm. After curing for 28 days, the concrete specimens were taken out to dry, and the surfaces of the concrete specimens were cleaned. The plastering work was carried out on the six surfaces of the concrete specimens in sequence. After the initial setting of the plaster mortar on the side surfaces, plaster layers on the top and bottom surfaces were added to complete the plaster on all the six surfaces of concrete specimens. After the plastering work was over, the concrete specimens were placed at room temperature for 2 weeks.

Their residual mass ratios, ultrasonic pulse velocities and various compressive strengths after heating were measured. In order to reveal the reasons of concrete degradation at different high temperatures, XRD diffractograms, scanning electron microscopy (SEM) images and thermogravimetric results of the concrete specimens were analyzed.

### 2.2. Test Materials

The OPC was of type CEM I 42.5R obtained from China Resources Cement Co., LTD. Foshan natural river sand with maximum diameter of 1.3 mm was used as fine aggregate, basalt with particle size of 5–30 mm was used as coarse aggregate. The water-reducing agent supplied from Shaanxi Qinfen building materials Co., Ltd. (Weinan, China). The chemical compositions of the OPC were obtained through X-ray diffraction method as displayed in Table 3. The OPC and aggregates were both produced in Guangdong Province in China, the water reducing agent was produced in Shanxi Province in China.

### 2.3. Test Method

#### 2.3.1. Concrete Slump Test

The concrete was poured into the slump barrel three times. After each filling, the rammer was used to hit the barrel wall 25 times to tamp it. The slump barrel was filled with concrete and the surface concrete was leveled. The slump bucket was pulled and the slump was calculated by subtracting the height of the peak of the concrete behind the collapse from the height of the bucket (300 mm).

#### 2.3.2. Heating Test

An integrated resistance furnace oven produced by Shanghai Zheti Machinery Manufacturing Co., Ltd. (Shanghai, China) was used for heating test. The rated power of the resistance furnace oven was 12 kW and the rated temperature was 1050 °C. After curing, the specimens were placed in a resistance furnace oven and kept at a constant temperature for 2 h after the furnace oven was heated up to the target temperature (200, 400, 600 and 800 °C) at a rate of 10 °C /min. Then the resistance furnace oven was turned off and the specimens were taken out. After the specimens were cooled to room temperature, cracks in the concrete specimens were observed. The remaining mass ratios of the concrete specimens were calculated by weighing the mass. Follow-up tests were carried out after the specimens were left standing at room temperature for 7 days.

#### 2.3.3. Ultrasonic Pulse Velocity Test

Zbl-u510 non-metallic ultrasonic detector was used in the ultrasonic pulse velocity test. Technical specification for detecting strength of concrete by ultrasonic-rebound combined method (CECS 02-2005, Beijing, China) was adopted in the test.

#### 2.3.4. Concrete Compressive Strength Test

A concrete compressive strength test was carried out according to Standard for evaluation of concrete compressive strength (GB/T50107-2010, Beijing, China), and the loading rate was 0.8 MPa/s.

#### 2.3.5. X-ray Diffraction (XRD) Analysis

After the concrete compressive strength test was completed, some fragments in the core of the concrete specimens were taken out for subsequent analysis. In order to stop the hydration reaction of concrete fragments, we soaked the concrete fragments with anhydrous ethanol for 3 days. After soaking, the concrete fragments were placed in a drying oven to dry (heating temperature was 40 °C) to constant weight, and ground through a 75 μm sieve. The analysis conditions were 10°~60°, 40 kV and 40 mA. The XRD test was performed with a Rigaku X-ray powder diffractometer ULTIMA IV (Tokyo, Japan).

#### 2.3.6. SEM Observation

The treatment process of mortar fragments was the same as that in Section 2.3.5. The mortar fragments were sprayed with gold and placed under a scanning electron microscope for observation. HITACHI S-4800 (Tokyo, Japan) was adopted in this test.

#### 2.3.7. Thermogravimetric Analysis (TGA)

The treatment process of mortar fragments was the same as that in Section 2.3.5, and the samples were heated from 30 °C to 950 °C at a rate of 10 °C/min under a nitrogen atmosphere. The test was conducted by using NETZSCH TG209F3 (Selb, Germany).

## 3. Results

### 3.1. Concrete Slump Test

Concrete slump results are shown in the second column of Table 4. The slump of concrete was in the range of 98~115 mm, reaching the goal of stable workability.

### 3.2. Appearance of Concrete after Heating Test

The appearance of concrete in each group after the heating test is shown in Table 5. Liu pointed out that the main reason for the fire spalling of concrete is the accumulation of high temperature vapor pressure in capillary pores and temperature stress caused by a temperature gradient [12]. Liang has reported that the main reason for explosive spalling is the accumulation of vapor pressure [13]. To conclude, spalling and explosion of concrete are caused by thermal stress and high-temperature vapor pressure. When the tensile stress caused by temperature gradient and vapor is greater than the tensile strength of the concrete, spalling or explosion occur.

Table 5 shows that with the rise of temperature, the appearance of concrete deteriorated to different degrees, which included cracking, spalling and even explosion. When the temperature exceeded 400 °C, the plastering layer began to fall off.

In addition, the test photos also show that in addition to temperature, water/cement ratio and the plastering layer influenced the deterioration of concrete appearance. Under the condition of no plastering layer, the lower the water/cement ratio of concrete was, the easier it was for it to explode. The concrete with a water/cement ratio of 0.35 and 0.40 only cracked and spalled after heating. Kodur pointed out that the explosion and spalling of HSC is due to the low water/cement ratio [14]. Hence, the concrete with 0.30 water/cement ratio began to explode at 400 °C due to its dense interior.

The test photos also show that the plastering layer can effectively alleviate the appearance deterioration of concrete, and this phenomenon was more obvious with the decrease of the water/cement ratio. The plastering layer had no obvious effect on the appearance deterioration of concrete with a 0.40 water/cement ratio at high temperatures. When the water/cement ratio dropped to 0.35, the cracking degree of concrete with a plastering layer was lower than that of concrete without a plastering layer. When the water/cement ratio dropped to 0.30, the plastering layer reduced the cracking degree and inhibited the explosion of concrete. This is because the plastering layer can slow the rate of concrete heating, resulting in a decrease in temperature gradient and vapor pressure [17,18,19,20].

### 3.3. Residual Mass Ratio

**Figure 1:**
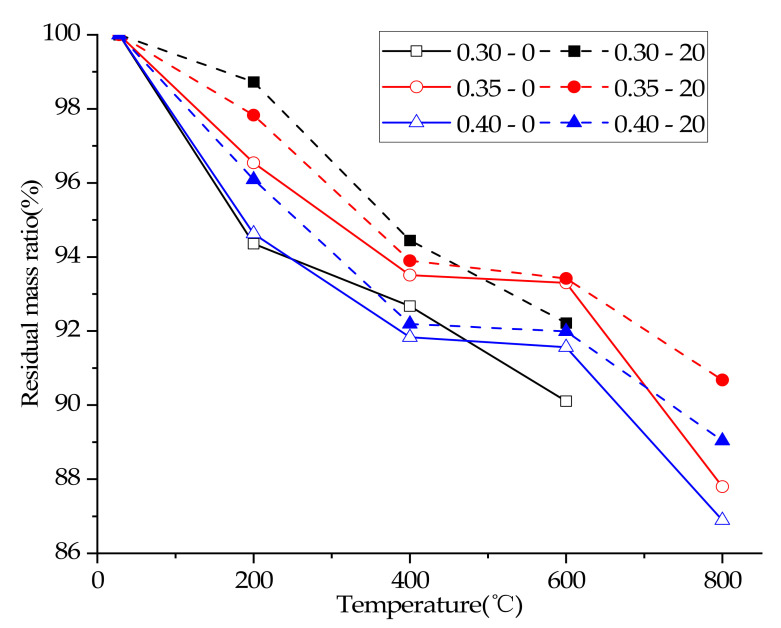
Residual mass ratio of concretes.

The ratio of residual mass of concrete of each group is shown in Table 4 and Figure 1. The residual mass ratio of concrete decreased significantly with the rise of temperature, which was caused by the loss of free water and chemically bound water [23,24,25]. (Note: there was no weighing calculation in this test when the concrete partially exploded).

At the same time, the data in Table 4 and Figure 1 also show that the plastering layer changed the residual mass ratio of concrete, because the plastering layer can delay the temperature rise of internal concrete [17,18,19,20] and thus the evaporation of free water of concrete. In addition, it was believed that under high temperature conditions, the plastering layer will act as a barrier to water vapor loss, and the water vapor will promote the continuous hydration of cement particles of concrete, which eventually reduced the free water overflow of concrete and increased the residual mass ratio.

Theoretically, the lower the water/cement ratio is, the denser the concrete is, and the less the free water is, which leads to the increase of the residual mass ratio of concrete after heating. However, in this test, the residual mass ratio of concrete at different temperatures increased first and then decreased with the increase of water/cement ratio. When the water/cement ratio dropped to 0.30, the residual mass ratio of concrete decreased, which was obviously in contradiction with the theory. When the water/cement ratio dropped to a certain extent, the concrete was more likely to crack and explode at high temperatures. When the water/cement ratio dropped to 0.30, concrete exploded at 400 °C, while concrete with 0.35 and 0.40 water/cement ratio had no obvious explosion (see Table 5 for details). The hypothesis was put forward: most of the concrete specimens with 0.30 water/cement ratio have slight cracking or spalling, which eventually led to the re-decline of the residual mass ratio when the water/cement ratio dropped to 0.30.

### 3.4. Ultrasonic Pulse Velocity

**Figure 2:**
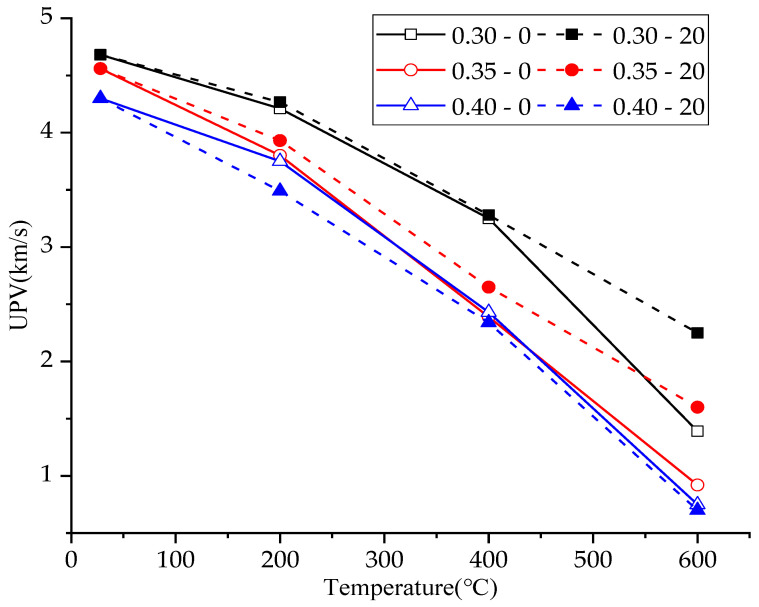
Ultrasonic pulse velocity of concretes.

Table 4 and Figure 2 show the specific values of ultrasonic pulse velocity after each group of concrete experienced heating. The vapor pressure, thermal stress and thermal decomposition of hydration products under high temperature led to the continuous development of concrete cracks. Therefore, the ultrasonic pulse velocity of concrete continued to decline with the rise of temperature. This is consistent with the conclusion of previous researchers. Bui mentioned that the UPV value of concrete decreased after exposure to 500 °C, no matter what mineral admixture was added to concrete [26]. By analyzing the test results, Lin found that residual UPV decreased with the increase of exposure temperature [27]. Uysal reported that the UPV of the heated SCC specimens decreased as the temperature increased, and there was a notable reduction in UPV shortly after the specimens were subjected to elevated temperatures greater than 400 °C [28]. Omer pointed out that with the increase of temperature, the UPV value of the sample decreased [29]. When the heating temperature reached 800 °C, obvious cracks appeared in all concrete specimens, and the specific ultrasonic pulse velocity could not be measured.

The data show that under the same temperature, the lower the water/cement ratio, the higher the ultrasonic pulse velocity of concrete, which can be explained from two aspects. The smaller the water/cement ratio was, the denser the concrete was, and the higher the ultrasonic pulse velocity was. In addition, with the decrease of water/cement ratio under normal temperature curing, more cement particles of concrete were not hydrated, which means that concrete can continue to be hydrated under high temperature and delay the decrease of ultrasonic pulse velocity caused by high temperature.

Besides temperature and water/cement ratio, the plastering layer also affected ultrasonic pulse velocity. When the water/cement ratio of concrete was 0.30 or 0.35, the plastering layer could act as a barrier to reduce the heating rate of the internal concrete [17,18,19,20] and delay the release of water vapor, providing conditions for the continued hydration of unhydrated cement particles of concrete. Therefore, the ultrasonic pulse velocity of concrete with a water/cement ratio of 0.30 or 0.35 and a plastering layer after high temperature exposure was higher than that of concrete without a plastering layer. When the water/cement ratio of concrete was 0.40, because most cement particles had been hydrated, high temperature did not promote cement hydration but could only cause the deterioration of concrete. In the process of natural cooling of concrete, the plastering layer significantly delayed the cooling process, resulting in the concrete with plastering layer being more likely to deteriorate due to high temperatures. Therefore, when the water/cement ratio was 0.40, the ultrasonic pulse velocity of the concrete specimens with a plastering layer, after experiencing high temperature, was lower than that of the concrete without a plastering layer.

### 3.5. Compressive Strength

**Figure 3:**
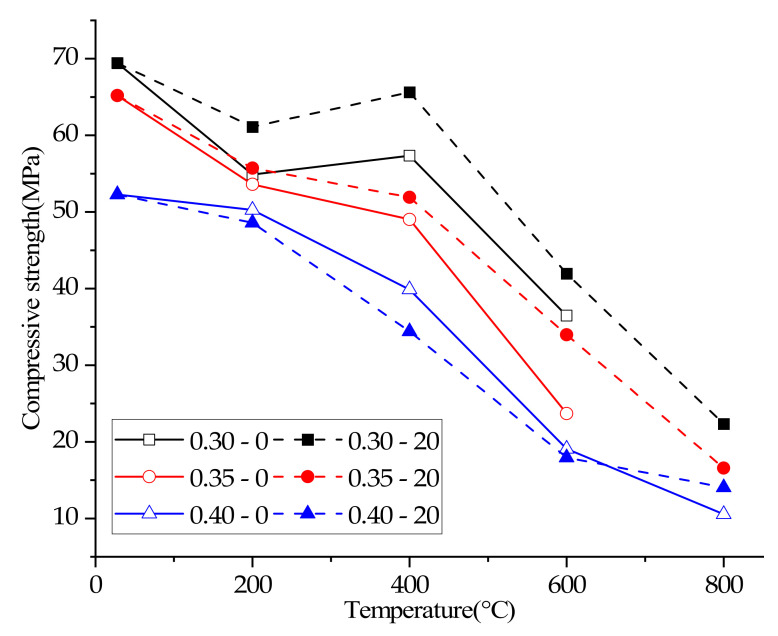
Compressive strengths of concrete samples.

Researchers pointed out that the residual UPV of concrete exposed to high temperature has a high correlation coefficient with the residual compressive strength, so the residual ultrasonic pulse velocity can be used to predict the residual compressive strength of concrete [26,27,29]. Therefore, it can be seen from Table 4 and Figure 3 that the decline law of concrete compressive strength is generally consistent with the decline law of ultrasonic pulse velocity. The plastering layer, water/cement ratio and plastering layer were all important factors affecting the compressive strength of concrete at different temperatures (because the damage of 0.30–0–800 °C and 0.35–0–800 °C was too serious to conduct a compressive strength test. This test did not test the compressive strength of the two groups of concrete).

The compressive strength of concrete with a water/cement ratio of 0.35 or 0.40 continued to decrease with the increase in temperature, regardless of whether there was a plastering layer. When the water/cement ratio was 0.30, the compressive strength of concrete decreased first, then rose, and then fell again with the increase of temperature. This phenomenon may be attributed to two reasons: (i) With the decrease of water/cement ratio, the number of unhydrated cement particles of concrete increased [30,31], and high temperature promoted the continuous hydration of unhydrated cement particles [3], so concrete with the lowest water/cement ratio (0.30) can realize the improvement of compressive strength in high temperatures. (ii) The improvement of compressive strength at high temperature can be attributed to the Van der Waals force [28,32].

In addition, it can be seen from Table 4 and Figure 3 that the plastering layer can enhance the compressive strength of concrete with a 0.30 water/cement ratio at 400 °C, which indicates that the plastering layer can delay the loss of free water and provide favorable conditions for high temperature rehydration of concrete with a low water/cement ratio.

### 3.6. XRD

**Figure 4:**
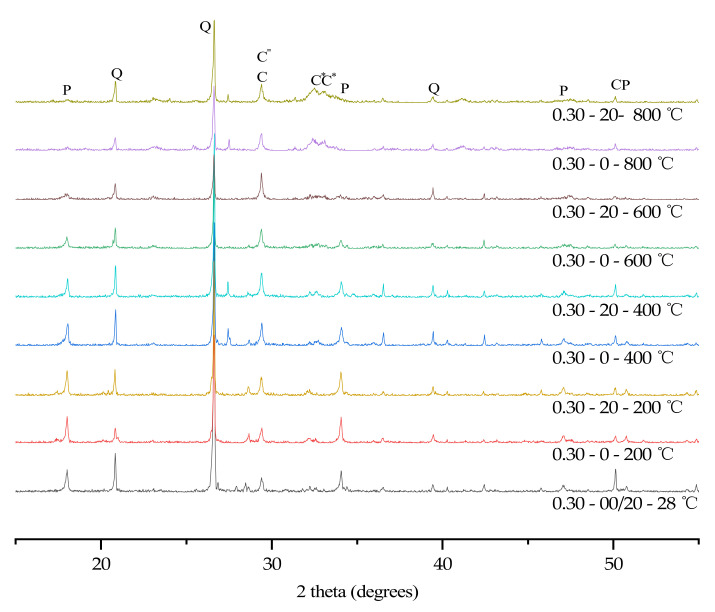
X-ray diffractograms of concrete (W/C = 0.30). Keys to phases: P (Portlandite); Q (Quartz); C (C-S-H gel); C″ (Calcite); C* (Calcium silicate).

**Figure 5:**
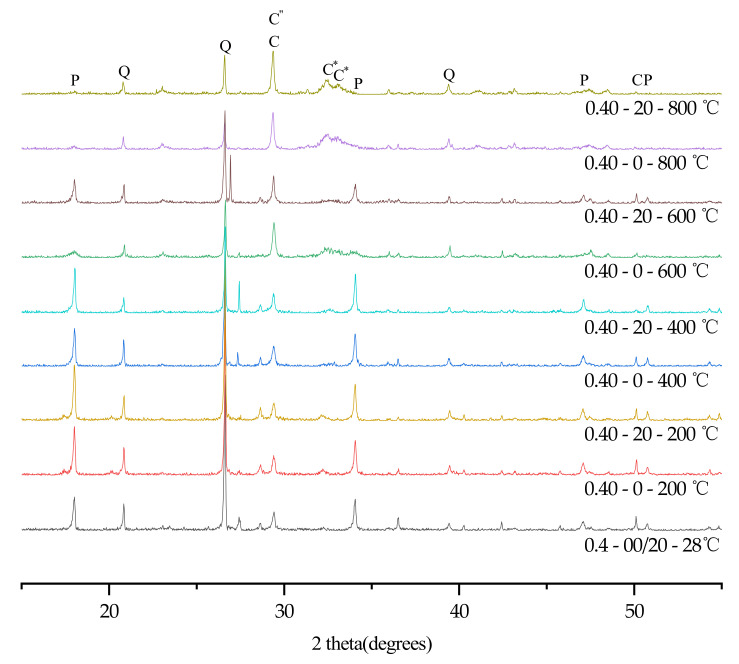
X-ray diffractograms of concrete (W/C = 0.40). Keys to phases: P (Portlandite); Q (Quartz); C (C-S-H gel); C″ (Calcite); C* (Calcium silicate).

In order to further analyze the influence of temperature, water/cement ratio and plastering layer on the hydration products of concrete, XRD tests were conducted on all specimens with a water/cement ratio of 0.30 and 0.40. The specific test results are shown in Figure 4 and Figure 5.

Because quartz has good high temperature resistance, the peak intensity of Quartz is very stable at high temperatures. High temperatures can promote the continuous hydration of unhydrated cement particles to generate CH. When the temperature exceeded 400 °C, CH rapidly decomposed [33]. Therefore, the content of CH reached its maximum strength around 400 °C [3]. As the temperature rose, the concrete cracked more rapidly, and a large amount of carbon dioxide in the air reacted with CH and calcium oxide to generate calcium carbonate. The content of calcium carbonate reached its maximum value at 600 °C, and when the temperature exceeded 700 °C, calcium carbonate decomposed in a large amount [34]. However, as the specimens stood still for 7 days, calcium oxide in the specimen reacted with carbon dioxide to generate calcium carbonate again. As C-S-H gel was dehydrated at a high temperature to generate calcium silicate [3,32], the content of dicalcium silicate continued to rise when the temperature exceeded 400 °C.

### 3.7. SEM

At room temperature, with the increase of water/cement ratio, the microstructure of concrete became sparser and a large amount of ettringite was generated. Hydration products of concrete with different water/cement ratios decomposed to different degrees at high temperatures: Ettringite disappeared at 200 °C. With the temperature rise, C-S-H gel and CH decomposed seriously at 600 °C. Moghadam found beta-Ca_2_SiO_4_ in an SEM figure of mortar heated at 800 °C [3]. Aydin observed crystals with rounded shapes suspected to be beta-Ca_2_SiO_4_ in an SEM figure of mortar heated at 900 °C, and pointed out that beta-Ca_2_SiO_4_ was the product of thermal decomposition of C-S-H gel at high temperature [32]. Therefore, the circular fine components of concrete after heating, might be beta-Ca_2_SiO_4_ produced by the thermal decomposition of C-S-H gel, which is consistent with the XRD results. This explains why concrete basically loses its compressive strength after 800 °C.

**Figure 6:**
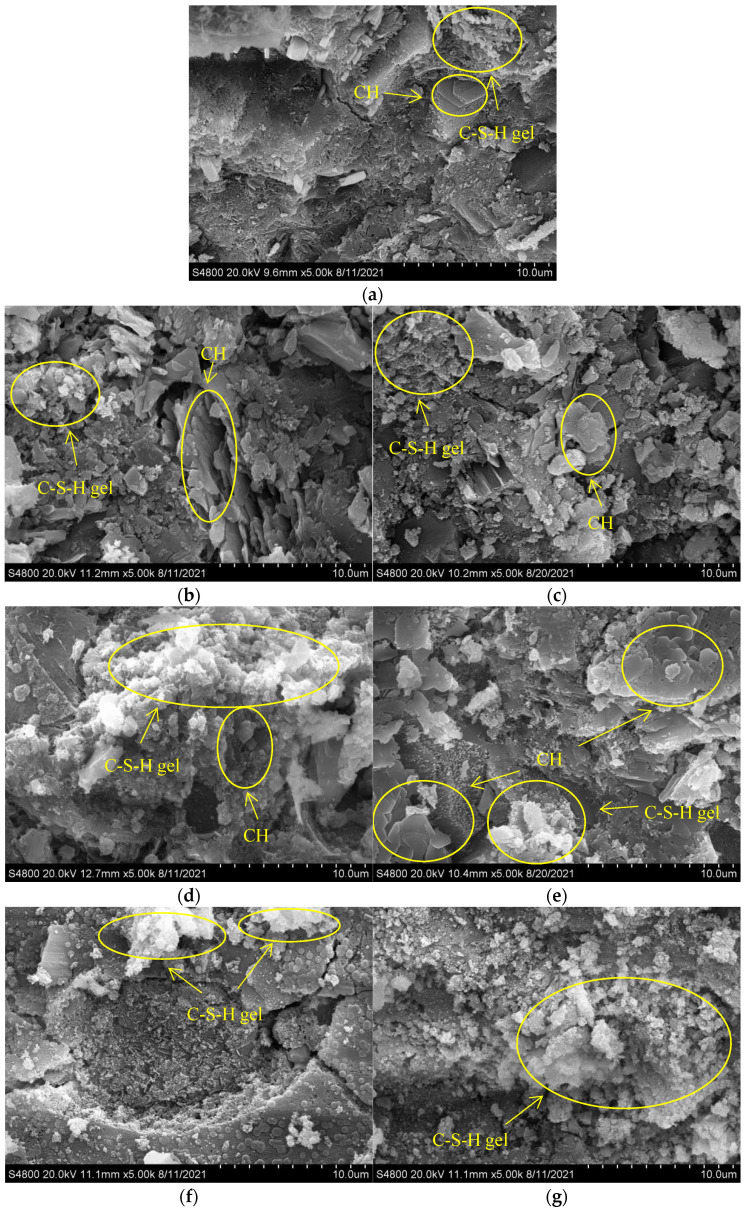

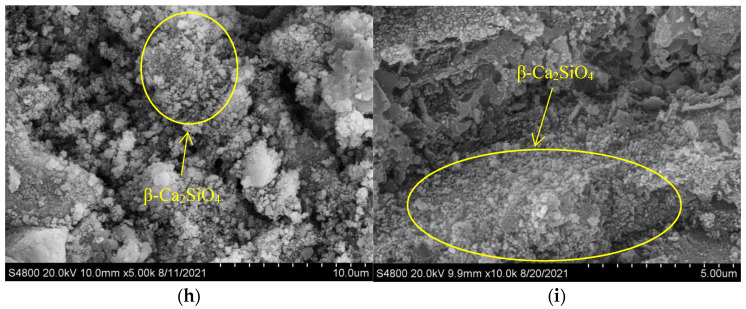
SEM analysis of concrete (water/cement ratio of 0.30). (**a**) 0.30–0/20–28 °C, (**b**) 0.30–0–200 °C, (**c**) 0.30–20–200 °C, (**d**) 0.30–0–400 °C, (**e**) 0.30–20–400 °C, (**f**) 0.30–0–600 °C, (**g**) 0.30–20–600 °C, (**h**) 0.30–0–800 °C, (**i**) 0.30–20–800 °C.

**Figure 7:**
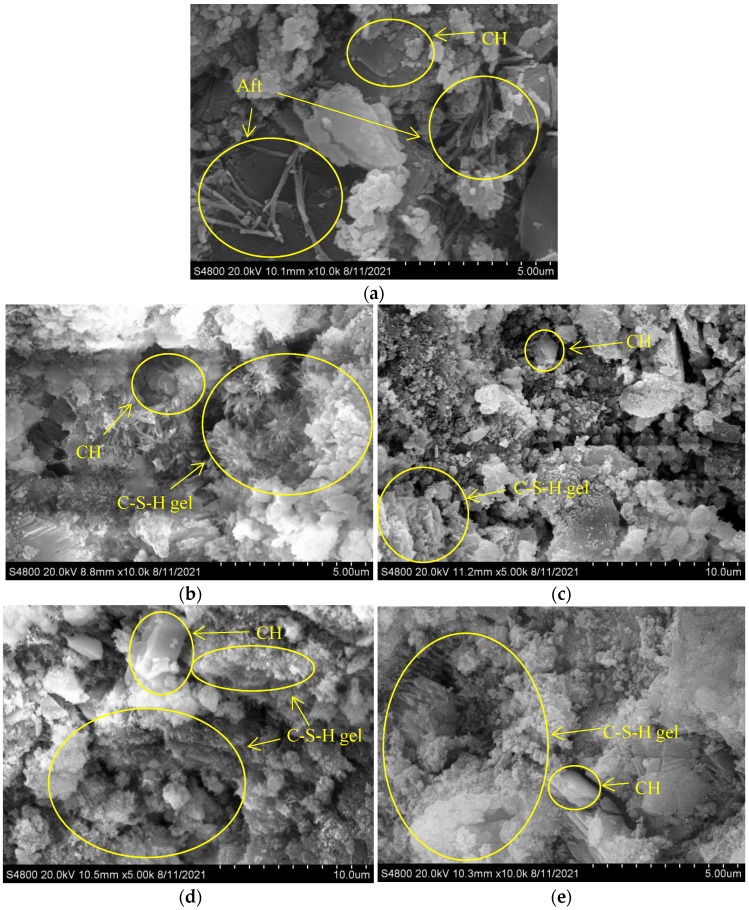

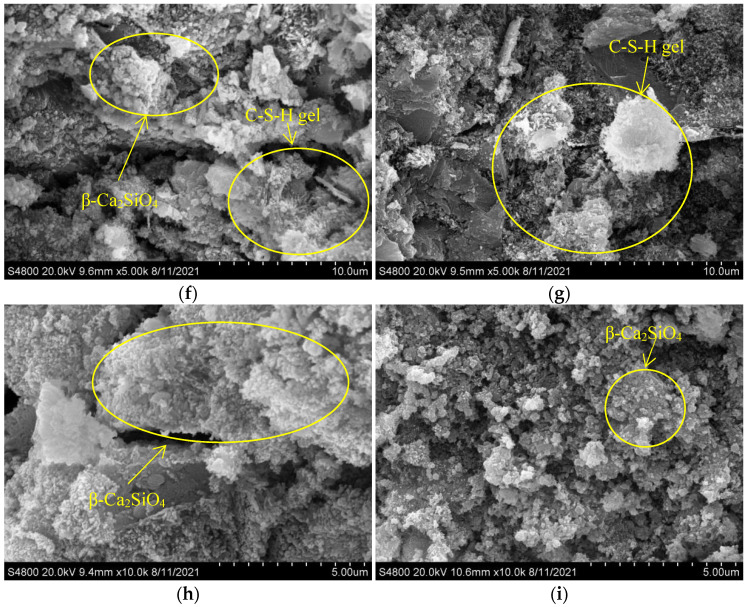
SEM analysis of concrete (water/cement ratio of 0.40). (**a**) 0.40–0/20–28 °C, (**b**) 0.40–0–200 °C, (**c**) 0.40–20–200 °C, (**d**) 0.40–0–400 °C, (**e**) 0.40–20–400 °C, (**f**) 0.40–0–600 °C, (**g**) 0.40–20–600 °C, (**h**) 0.40–0–800 °C, (**i**) 0.40–20–800 °C.

By comparing Figure 6 and Figure 7, it can be found that the concrete with large water/cement ratio not only presented more sparse microscopic morphology at room temperature, but also presented more sparse microscopic morphology at high temperatures, which was consistent with the previous ultrasonic pulse velocity test results and compressive strength test results.

Although the previous ultrasonic pulse velocity test results and XRD test results show that the plastering layer changed the crack development behavior and hydration product decomposition behavior of concrete at high temperatures, this was not clearly reflected in the SEM figure, which may be the result of the error caused by the selection of points in the test.

### 3.8. TGA

**Figure 8:**
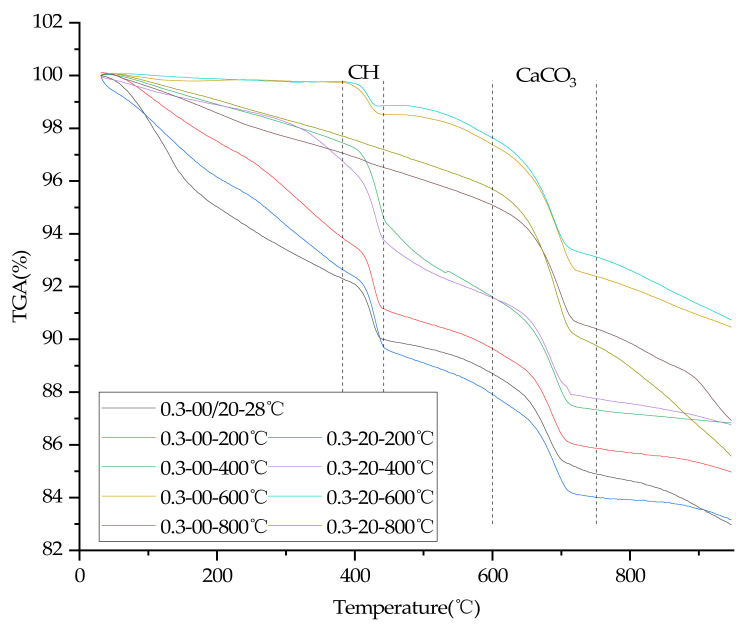
TGA test results of concrete specimens (water/cement ratio of 0.30).

**Figure 9:**
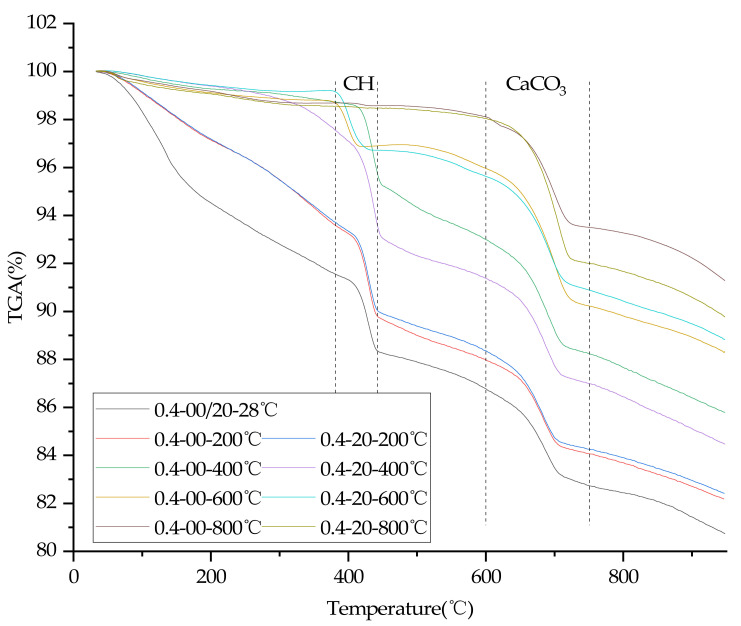
TGA test results of concrete specimens (water/cement ratio of 0.40).

In order to quantify the effects of temperature, water/cement ratio and plastering layer on the contents of C-S-H gel of concrete, thermogravimetric tests were carried out in this study. Figure 8 and Figure 9 are TGA diagrams of concrete with 0.3 and 0.4 water/cement ratio, respectively.

The C-S-H gel amount of the sample were calculated according to Equation (1) [35], C-S-H gel ratio were calculated according to Equation (2).

(1) $$C-S-H\;\mathrm{gel}\left(\%\right)\;=\mathrm{TotalLOI}-\mathrm{LOICH}-{\mathrm{LOICaCO}}_{3}$$

where TotalLOI is the ratio of mass loss of sample, LOICH and LOICaCO3 refer to the ratio of mass loss due to the decomposition of CH (around 400 °C [3]) and CaCO3 (600–750 °C [35]).

(2) $$C-S-H\;\mathrm{gel}\;\mathrm{ratio}\left(\%\right)\;=C-S-H\;\mathrm{gel}\left(\mathrm{WP}\right)/C-S-H\;\mathrm{gel}\left(\mathrm{WOP}\right)\;$$

where C-S-H gel(WP) is the amount of C-S-H gel of concrete with a plastering layer, and C-S-H gel(WOP) is the amount of C-S-H gel of concrete without a plastering layer.

**Figure 10:**
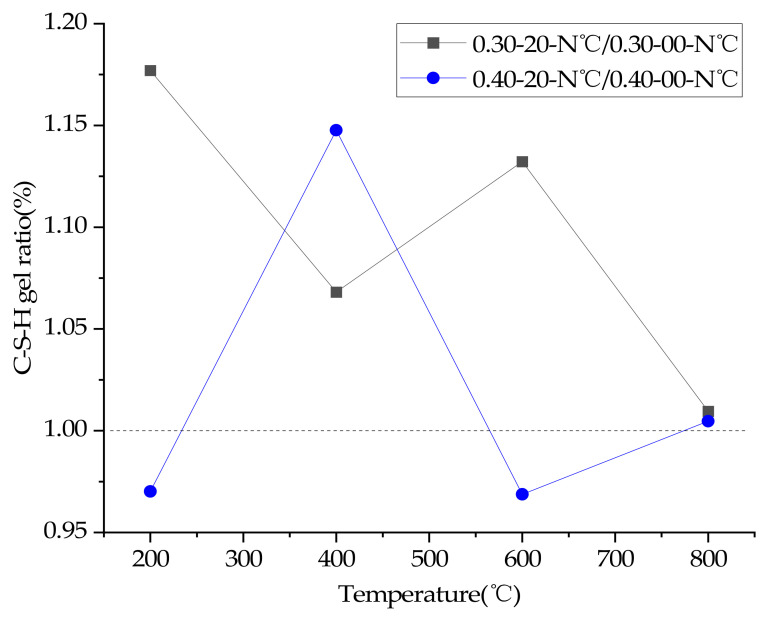
C-S-H gel content ratio of concrete specimens.

The calculation results of C-S-H gel ratio are shown in Figure 10. It can be seen from Figure 10 that the C-S-H content of concrete with a 0.3 water/cement ratio and a plastering layer is higher than that of concrete without a plastering layer after heating. However, after heating at different temperatures, the C-S-H gel content of 0.4 water/cement ratio concrete with a plastering layer is not significantly different from or even slightly lower than its counterpart without a plastering layer (except at 400 °C). The result indicates that a plastering layer can increase the C-S-H gel content of 0.3 water/cement ratio concrete at high temperatures by promoting the hydration of unhydrated cement particles. However, the C-S-H gel content of 0.4 water/cement ratio concrete cannot be improved significantly under the action of a plastering layer at high temperatures, such as that with a 0.3 water/cement ratio concrete, and the C-S-H gel content of 0.4 water/cement ratio concrete even drop slightly after heating at 200 °C and 400 °C under the action of the plastering layer.

## 4. Discussion of Results

Temperature is an important factor leading to the deterioration of concrete, as high temperatures will bring high-temperature vapor pressure, thermal stress and thermal decomposition of the hydration products and other adverse factors [2,3,4,5,6,7], and eventually lead to the deterioration of the appearance of concrete (cracking, spalling or even explosion) and compressive strength loss.

Although the compressive strength of concrete increased with the decrease of the water/cement ratio at all temperatures, the concrete with a low water/cement ratio (0.30) was too dense, which led to the agglomeration of water vapor and easily led to explosion.

The test results show that the factors that changed the high temperature resistance of concrete were not only the water/cement ratio but also the plastering layer, and whether the plastering layer improved the high-temperature resistance of concrete depended on the water/cement ratio of concrete. For concrete with a high water/cement ratio, most cement was hydrated during the 28 days curing [30,31], so the concrete lacked reactants to continue hydration at high temperatures. When the water/cement ratio dropped, the cement of the concrete was not completely hydrated during the 28 days curing, and the compressive strength of the concrete even increased at 400 °C due to the Van der Waals force [28,32] and hydration of the unhydrated cement.

There were two main functions of the plastering layer in the heating test: reducing the rate of rise and drop of the temperature of internal concrete [17,18,19,20], and delaying the loss of high-temperature water vapor. Under the condition of high temperature, water vapor can promote the rapid hydration of unhydrated cement particles [3,4,5]. However, due to the lack of unhydrated cement particles of concrete with a 0.40 water/cement ratio, high temperature did not improve the compressive strength of concrete with a 0.40 water/cement ratio. Analysis of the thermogravimetric test results showed that a plastering layer could accelerate the decomposition of C-S-H gel since the plastering layer delayed the cooling of concrete, eventually leading to the decline of compressive strength of concrete with a 0.4 water/cement ratio.

Analysis of the thermogravimetric tests results also proved that concrete with a water/cement ratio of 0.30 at high temperatures can continue to hydrate unhydrated cement particles to generate more C-S-H gel, as the plastering layer slows the escape of water vapor, resulting in a slower rate of compressive strength decline of concrete (and even compressive strength grew at 400 °C). In addition, the plastering layer can completely inhibit the cracking of 0.30 water/cement ratio concrete at high temperatures, because the plastering layer can delay the heating rate of internal concrete and thus delay the damage of high temperature vapor pressure and thermal stress on concrete.

Generally speaking, concrete will deteriorate with the rise of temperature, which leads to the decline of compressive strength. At the same time, the test results also show that the compressive strength of concrete after heating is affected by the water/cement ratio and the plastering layer. Therefore, the influence of the water/cement ratio and plastering layer on concrete compressive strength after heating should be considered when calculating the fire resistance limit and residual load capacity of concrete members, and the compressive strength of heated concrete should not be calculated directly by a reduction factor.

## 5. Conclusions

Through analyzing the appearance of concrete after exposure to high temperatures, ultrasonic pulse velocity, compressive strength and hydration products, the main conclusions are as follows:

(1) Reducing the water/cement ratio can improve the strength of concrete at any temperature, but too low water/cement ratio can easily cause high temperature cracking, and ultimately lead to the decline of high temperature resistance of concrete. Under the condition of no plastering layer, all concrete with a water/cement ratio of 0.3 or 0.35 cannot be tested for compressive strength by spalling and explosion at 800 °C. Concrete with a water/cement ratio of 0.4 can still be tested for compressive strength despite obvious cracking.
(2) After exposure to a high temperature (400 °C), the compressive strength of 0.30–0–400 °C was 4.48% higher than 0.30–0–200 °C, and the compressive strength of 0.30–20–400 °C was 7.38% higher than 0.30–20–200 °C. The increase of compressive strength can be attributed to the hydration of unhydrated cement particles of concrete and the Van der Waals force.
(3) A plastering layer can accelerate or decelerate the damage of concrete at high temperatures, which depends on the water/cement ratio of concrete. A plaster layer at a high temperature can inhibit water vapor’s escape to promote the hydration of unhydrated cement in concrete with a low water/cement ratio (0.3) to delay the loss of the compressive strength of concrete. However due to the high hydration rate of concrete with a high water/cement ratio (0.4) the plaster layer cannot delay the loss of the compressive strength of concrete at high temperatures and can even exacerbate the loss of the compressive strength of concrete. Therefore, the plastering layer can increase the compressive strength of concrete with a 0.3 water/cement ratio by 11.35–15.02% between 200 °C and 800 °C and increase the compressive strength of concrete with a 0.35 water/cement ratio by 3.95–43.33% between 200 °C and 800 °C. However, the plastering layer decreases the compressive strength of concrete with 0.4 water/cement ratio between 200–600 °C, and increases the compressive strength by 33.40% at 800 °C.
(4) The compressive strength of concrete after heating is affected by the water/cement ratio and plastering layer, so the influence of the water cement/ratio and plastering layer should be considered when calculating the fire resistance limit and residual load capacity of concrete members under fire, and the reduction factor should not be directly used to calculate the strength of concrete (especially concrete with a low water/cement) after heating.

## Figures and Tables

**Table 1:** Mix proportion of concrete.

Mix No.	W/C Ratio	Cement (kg/m^3^)	Water (kg/m^3^)	Fine Aggregate (kg/m^3^)	Coarse Aggregate (kg/m^3^)	Water Reducer (g/m^3^)
0.30–0–25 °C	0.30	633.8	190.1	720.9	917.4	3300
0.30–0–200 °C						
0.30–0–400 °C						
0.30–0–600 °C						
0.30–0–800 °C						
0.30–20–25 °C						
0.30–20–200 °C						
0.30–20–400 °C						
0.30–20–600 °C						
0.30–20–800 °C						
0.35–0–25 °C	0.35	587.3	205.5	720.9	917.4	2000
0.35–0–200 °C						
0.35–0–400 °C						
0.35–0–600 °C						
0.35–0–800 °C						
0.35–20–25 °C						
0.35–20–200 °C						
0.35–20–400 °C						
0.35–20–600 °C						
0.35–20–800 °C						
0.40–0–25 °C	0.40	547.1	218.8	720.9	917.4	400
0.40–0–200 °C						
0.40–0–400 °C						
0.40–0–600 °C						
0.40–0–800 °C						
0.40–20–25 °C						
0.40–20–200 °C						
0.40–20–400 °C						
0.40–20–600 °C						
0.40–20–800 °C						

**Table 2:** Mix proportion of the mortar for plastering.

Cement (kg/m^3^)	Water (kg/m^3^)	Fine Aggregate (kg/m^3^)
540.0	297.0	1350.0

**Table 3:** Chemical compositions of the OPC.

Chemical Composition	OPC (%)
SiO_2_	23.9
CaO	62.3
Al_2_O_3_	4.9
Fe_2_O_3_	3.7
MgO	2.4
Na_2_O	<0.3
K_2_O	0.4
Loss on ignition	1.9

**Table 4:** Test results of each concrete mixture.

Mix No.	Slump of Concrete (mm)	Residual Mass Ratio (%)	Ultrasonic Pulse Velocity (km/s)	Compressive Strength (MPa)
0.30–0–25 °C	115	100%	4.68	69.43
0.30–0–200 °C	115	94.36%	4.21	54.89
0.30–0–400 °C	115	92.67%	3.25	57.35
0.30–0–600 °C	115	90.11%	1.39	36.48
0.30–0–800 °C	115	no	no	no
0.30–20–25 °C	115	100%	4.68	69.43
0.30–20–200 °C	115	98.73%	4.26	61.12
0.30–20–400 °C	115	94.45%	3.28	65.63
0.30–20–600 °C	115	92.22%	2.25	41.96
0.30–20–800 °C	115	no	no	22.36
0.35–0–25 °C	98	100%	4.56	65.20
0.35–0–200 °C	98	96.54%	3.80	53.61
0.35–0–400 °C	98	93.51%	2.39	49.03
0.35–0–600 °C	98	93.30%	0.92	23.70
0.35–0–800 °C	98	87.80%	no	no
0.35–20–25 °C	98	100%	4.56	65.2
0.35–20–200 °C	98	97.83%	3.93	55.73
0.35–20–400 °C	98	93.90%	2.65	51.92
0.35–20–600 °C	98	93.42%	1.60	33.97
0.35–20–800 °C	98	90.68%	no	16.58
0.40–0–25 °C	110	100%	4.30	52.29
0.40–0–200 °C	110	94.63%	3.75	50.27
0.40–0–400 °C	110	91.83%	2.34	39.89
0.40–0–600 °C	110	91.56%	0.75	19.09
0.40–0–800 °C	110	86.89%	no	10.54
0.40–20–25 °C	110	100%	4.30	52.29
0.40–20–200 °C	110	96.09%	3.49	48.60
0.40–20–400 °C	110	92.19%	2.43	34.41
0.40–20–600 °C	110	91.99%	0.70	17.95
0.40–20–800 °C	110	89.04%	no	14.06

**Table 5:** Appearance of concrete specimens after high temperature resistance test.

Groups	With Mortar Plastering Layer	Without Mortar Plastering Layer
0.30–0(20)–200 °C	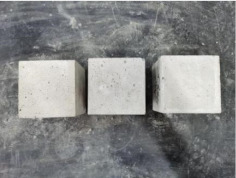	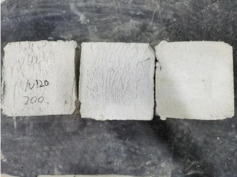
0.30–0(20)–400 °C	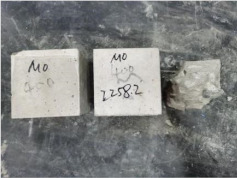	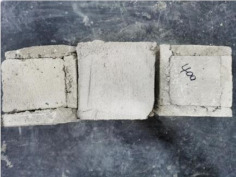
0. 30–0(20)–600 °C	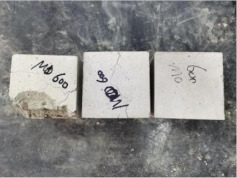	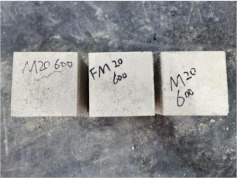
0.30–0(20)–800 °C	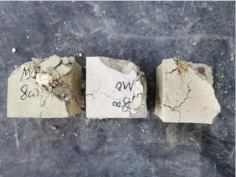	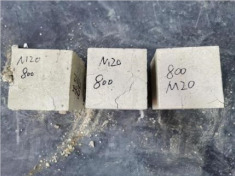
0.35–0(20)–200 °C	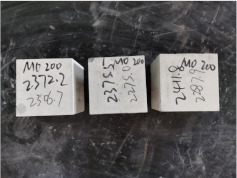	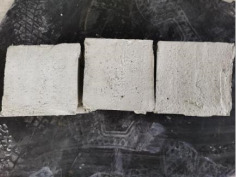
0.35–0(20)–400 °C	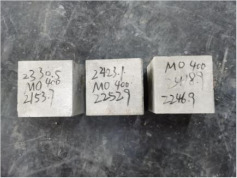	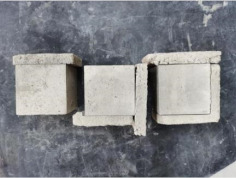
0.35–0(20)–600 °C	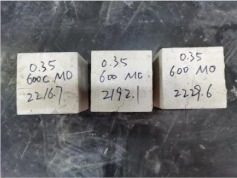	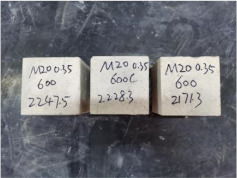
0.35–0(20)–800 °C	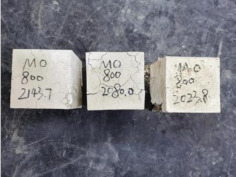	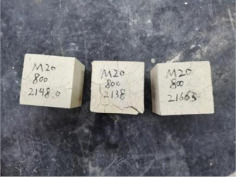
0.40–0(20)–200 °C	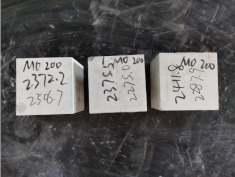	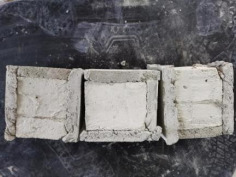
0.40–0(20)–400 °C	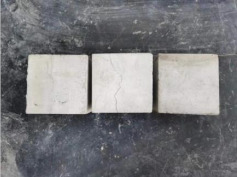	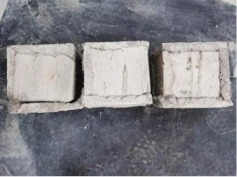
0.40–0(20)–600 °C	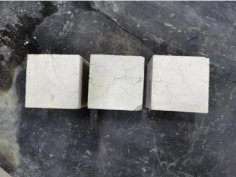	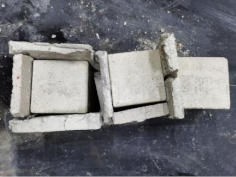
0.40–0(20)–800 °C